# Recent Advances and Perspectives in Anesthesiology: Towards Artificial Intelligence-Based Applications

**DOI:** 10.3390/jcm13154316

**Published:** 2024-07-24

**Authors:** Marco Cascella, Massimo Antonio Innamorato, Alessandro Simonini

**Affiliations:** 1 Department of Medicine, Surgery and Dentistry, University of Salerno, 84081 Baronissi, Salerno, Italy; 2 Pain Unit, Department of Neuroscience, Santa Maria delle Croci Hospital, AUSL Romagna, 48121 Ravenna, Italy; massimo.innamorato@auslromagna.it; 3 Pediatric Anesthesia and Intensive Care Unit, Salesi Children’s Hospital, 60123 Ancona, Italy; dr.simonini@gmail.com

In recent years, the field of anesthesiology has seen remarkable advancements in patient safety, comfort, and outcomes. New drugs, technologies, techniques, and pathways have been developed and investigated. For example, enhanced recovery after surgery (ERAS) protocols are integrated multimodal treatment processes designed to achieve early recovery for patients undergoing major surgery [1,2]. ERAS applications reflect a collaborative effort across multiple disciplines and encompass advanced anesthesia strategies, such as ultrasound-guided regional anesthesia techniques [3]. Perioperative monitoring has also seen significant enhancements with sophisticated monitoring systems [4]. Additionally, the integration of closed-loop systems, which automatically adjust anesthetic delivery based on real-time monitoring data, has further optimized anesthesia management and patient safety [5].

This special issue on recent advances in anesthesiology was designed to bring together cutting-edge research and expert insights that underscore the dynamic evolution of this critical medical specialty. Therefore, the five articles featured in this issue highlight innovative strategies and interventions that can contribute to enhancing perioperative care and improving patient outcomes.

Briefly, this special issue addresses selected topics, from innovative pain management techniques to enhanced patient safety measures and personalized treatment approaches. Moreover, special attention has been devoted to the chapter of chronic pain. Given the current opioid crisis, where opioid addiction and overdose deaths are major public health issues, the potential for postoperative patients to develop dependency or engage in misuse is a serious concern. Notably, surgical opioid stewardship initiatives are suggested [6]. In this complex scenario, one of the articles of the collection, by Neuschmid et al. (Contributor 1), concerns the topic of postoperative pain following video-assisted thoracoscopic surgery. This retrospective analysis demonstrates that the use of intercostal catheters (ICC) combined with a single-shot intraoperative intercostal nerve block significantly reduces opioid consumption (*p* = 0.023) and the incidence of chronic post-surgery pain (CPSP) one year after surgery (*p* = 0.035). The findings suggest that ICC can provide superior pain relief, leading to a lower frequency and duration of opioid use. This is further proof indicating that a multimodal careful strategy can be crucial in mitigating the risk of opioid dependency and enhancing overall patient recovery.

The processes that define perioperative pathways are becoming increasingly structured; however, changes in clinical practices necessitate definite educational programs focused on quality improvement. These aspects closely concern perioperative management. According to the National Center for Complementary and Integrative Health, nonpharmacological pain management strategies encompass any intervention that helps reduce pain without relying on medication [7]. In another compelling contribution, Salamon et al. (Contribution 2) explore the implementation of nonpharmacological interventions for pain management in pediatric inpatients. The pilot project study on quality improvement highlights the challenges of integrating these strategies, such as the lack of awareness and the complexity of the referral process. The authors found that the introduction of a best practice alert developed to trigger a message after a patient indicated a level 5 or higher pain score (on a 0–10 scale) after two separate vital checks within the electronic medical record system resulted in a significant increase in the utilization of these services. These preliminary results underscore the potential of systemic changes to improve pain management practices in different surgical settings.

In terms of patient safety, the collection also delves into the persistent challenge of residual neuromuscular block (RNMB) after emergence, as discussed in Blum et al.’s comprehensive review (Contribution 3). This article emphasizes the under-recognition of RNMB and its association with adverse outcomes such as postoperative pulmonary complications and prolonged hospital stays. The authors underscore the adoption of quantitative neuromuscular monitoring and the use of a neuromuscular block reversal, which can significantly enhance patient safety and satisfaction.

Due to the serious concern of the chronic disease epidemic and to address the negative impacts of chronic conditions, the European Commission launched the three-year (2017–2020) Joint Action for Chronic Disease Plus (CHRODIS Plus) initiative. Building on the previous Joint Action CHRODIS (2013–2017), which identified, validated, and implemented effective practices for managing chronic diseases across Europe, CHRODIS Plus aimed to further enhance health promotion, prevent chronic illnesses, improve care for patients with multiple conditions, and develop the CHRODIS Plus Workbox on Employment and Chronic Conditions (CPWEC) [8]. On these bases, addressing chronic pain from a broader perspective, Lilley et al. (Contribution 4) provide an appraisal of the CHRODIS Plus initiative in their scoping review. The study critically examines the evidence supporting workplace interventions for chronic conditions, revealing gaps in the quality of the underlying research. The paper highlights the need for robust post-implementation evaluations to validate the efficacy of such initiatives and ensure they are based on solid scientific foundations.

Finally, the state-of-the-art clinical perspective by Nijs et al. (Contribution 5) underscores the importance of addressing behavioral lifestyle factors in the management of chronic pain. This article synthesizes evidence on multimodal interventions that target physical activity, stress, sleep, and nutrition, advocating for a personalized approach that integrates these elements into comprehensive treatment plans. The growing body of evidence supporting this approach offers promising avenues for improving the quality of life for individuals suffering from chronic pain.

The articles cover just a few aspects of a highly diverse research landscape. The research prospects are exceptionally promising. They include, for instance, personalized anesthesia approaches through genetic and pharmacogenomic testing to understand how genetic variations affect drug metabolism and response [9]. This can help tailor anesthesia plans to individual patients, reducing the risk of adverse reactions. On the other hand, this strategy can help identify postoperative trajectories and the impact of anesthesia on disease-related outcomes [10,11].

Additionally, the perspectives encompass artificial intelligence (AI) approaches for predictive analytics and decision support systems [12]. These advanced technologies leverage machine learning (ML) algorithms to analyze large datasets and predict outcomes based on patterns and trends identified in the data. Consequently, AI-based decision support systems can provide clinicians with real-time insights and recommendations based on the latest evidence and patient-specific data. This is another pivotal step toward personalized and evidence-based care in anesthesia [13]. Furthermore, this capability not only aids in making informed decisions but also optimizes resource allocation and enhances overall healthcare delivery [14].

AI applications in anesthesia are not limited to ML; artificial neural architectures of varying complexity and for different purposes are investigated. These architectures range from simple feedforward neural networks to more advanced structures like convolutional neural networks (CNNs) and recurrent neural networks (RNNs), each offering unique capabilities for different aspects of anesthetic practice. A long Short-Term Memory (LSTM) network is a type of RNN specifically designed to learn and remember long-term dependencies and patterns in sequential data, which makes it particularly effective for tasks involving time series or sequences. Different attempts have been conducted in anesthesia. For example, Kim et al. [15] collected 18,163 pictures from 3053 patients and developed a CNN model to predict difficult direct laryngoscopy. Recently, Hu et al. [16] developed a deep learning model using CNN and LSTM architectures to predict anesthetic drug dosages during surgeries, addressing the challenge of individual physiological differences. The model incorporated a SELU activation function and weighted regularization to handle sample imbalance and a 7 × 1 convolution kernel with pooling techniques for feature extraction. The bidirectional LSTM network improved learning of patient state changes, and an attention module enhanced focus on key features. Regarding performance, the model significantly improved prediction accuracy, reducing the mean absolute percentage error (MAPE) to 15.83% for preoperative dosage predictions and 12.25% for intraoperative predictions. Additionally, the coefficient of determination (R^2^ values) increased to 0.887 and 0.915, respectively, indicating a higher proportion of variance in the data being explained by the model and thereby reflecting better predictive performance and reliability.

Interestingly, AI models can be used to tackle the increased use of postoperative opioids. Natural language processing, a subfield of AI, was implemented for this aim [17,18]. Furthermore, there is a place for innovative digital health technologies and telemedicine in anesthesia, mostly for preanesthesia assessment [19], and pain management [20,21]. Yet, the use of wearable devices seems to be suitable for postoperative monitoring in surgical wards [22]. Nevertheless, for achieving effective AI applications in healthcare, precise multidisciplinary strategies and critical ethical issues should be necessarily addressed [23]. A critical aspect of implementing AI in clinical practice is the need to validate various AI models in real-world scenarios. While these models may perform well in controlled, experimental settings, their effectiveness in actual clinical environments must be thoroughly assessed [24]. 

Finally, education and simulation strategies are being enhanced by implementing virtual reality (VR) and augmented reality (AR) technologies for training anesthesiologists in complex procedures and emergency scenarios, along with high-fidelity simulators that replicate real-life scenarios for hands-on practice without any risk to patients [25]. In conclusion, the adoption of AI technologies represents a significant stride towards advancing systems anesthesiology, aiming to improve intraoperative and perioperative management across different critical aspects of patient care including hemodynamic monitoring and pain management [26]. It is increasingly feasible to envision anesthesia providers soon utilizing sophisticated instruments that integrate biomarkers, genetic data, multidimensional assessments, and state-of-the-art technology.
